# Interacting with the public as a risk factor for employee psychological distress

**DOI:** 10.1186/1471-2458-10-435

**Published:** 2010-07-25

**Authors:** Michael F Hilton, Harvey A Whiteford

**Affiliations:** 1 University of Queensland, School of Population Health, Herston, QLD, Australia; 2 Queensland Centre for Mental Health Research, Wacol, QLD, Australia

## Abstract

**Background:**

The 1-month prevalence of any mental disorder in employees ranges from 10.5% to 18.5%. Mental disorders are responsible for substantial losses in employee productivity in both absenteeism and presenteeism. Potential work related factors contributing to mental difficulties are of increasing interest to employers. Some data suggests that being sales staff, call centre operator, nurse or teacher increases psychological distress. One aspect of these occupations is that there is an interaction with the public. The aim of this study is to evaluate whether employees who interact with the public are at greater risk of psychological distress.

**Methods:**

Data was collected from two studies. In study one 11,259 employees (60% female; mean age 40-years ± SD 10-years) from six employers responded to the Health and Work Performance Questionnaire (HPQ) which contained a measure of psychological distress, the Kessler 6 (K6). Employees were coded as to whether or not they interacted with the public. Binomial logistic regression was performed on this data to determine the odds ratio (OR) for moderate or high psychological distress in employees that interacted with the public. Study two administered the HPQ and K6 to sales employees of a large Australian bank (N = 2,129; 67% female; mean age 39-years SD 10-years). This questionnaire also probed how many contacts individuals had with the public in the past week. Analysis of variance was used to determine if the number of contacts was related to psychological distress.

**Results:**

In study one the prevalence of psychological distress in those that interacted and did not interact with the public were 19% and 15% respectively (P < 0.001). Interacting with the public was associated with an increased OR of 1.3 (P < 0.001) for moderate to high levels of psychological distress. In study two employees with less than 25 contacts with the public per week had a lower K6 score than those who had ≥ 25 contacts per week (P = 0.016).

**Conclusions:**

The results of the current study are indicative that interaction with the public increases levels of psychological distress. Employees dealing with the public may be an employee subgroup that could be targeted by employers with mental health interventions.

## Background

Mental disorders are prevalent in general society, including the working population. The recent Australian Survey of Mental Health and Well-being found that 17% of the general population had an anxiety or depressive disorder in the previous 12 months[1]. In Australia it is estimated that 10.5% of employees have a 1-month prevalence of any mental disorder[2]. Using data from employees in the US National Comorbidity Survey (NCS) study, Kessler et al. find that 18.2% of US employees have 1-month prevalence of any mental disorder[3]. Not only are mental disorders prevalent but they cause substantial losses in employee productivity[4-11]. It is therefore in the interest of employers, governments and policy makers to reduce the incidence and prevalence of mental health problems in the workplace. In this regard there have been numerous studies attempting to identify workplace factors that increase the risk of developing mental health problems in employees.

Stress and mental health symptoms and/or conditions have been shown to be problematic amongst nurses [12-15], teachers [16-19], call centre operators [20,21] and sales staff [3,22]. Encountering difficult clients was reported as the largest risk factor for job stress in call centre operators [21]. One element that all the aforementioned occupations have is that they all interact with the public. Thus, we postulated that employee interaction with the public may be a contributor to worsening employee mental health. To test this hypothesis in Study 1 employee data from the Work Outcomes Research Cost-benefit (WORC) project, described in the methods, was extracted. Prevalence rates of psychological distress in employee categories of dealing and not dealing with the public were examined. An additional study (Study 2), with data collected separately from the WORC study, was conducted in the sales force of a large Australian bank. This data examines the changes in psychological distress by the number of client interactions per week.

## Methods

### Sample

Data collected for this paper emanated from two separate studies. Firstly as part of the Work Outcomes Research Cost-benefit (WORC) Project, employees of 58 large (> 1,000 employees) Australian employers were presented with the World Health Organisation Health and Work Performance Questionnaire (HPQ). Detailed descriptions of the recruitment of employers and employees in the study have been previously published[22,23]. 60,556 full-time employees had valid responses to the HPQ (response rate 25%). Surveys were received between October 2004 and December 2005. Only employees over the age of 18-years were invited to participate. The HPQ included the Kessler 6 (K6) a brief survey quantifying psychological distress. Each individual employer in the WORC project provided employee categories meaningful to that specific employer. This was done so that an employer specific relevant report could be delivered to each employer on the mental health profile of their organisation. Of the 58 participating companies six included an employee classification of customer service, call centre or counter staff. These categories were defined as those employees who interacted with the public. In total the six employers included in the study (Study 1) had 11,259 employees (60% female; mean age 40-years SD 10-years). The prevalence of psychological distress in those employees who interacted with the public versus all other employees for these six employers was examined. The employers were from the telecommunications, utilities, local government, federal government and news media sectors. The percentage of employees identified in the interaction with the public category is located in Table 1 under the heading "% customer staff".

**Table 1:** Psychological distress prevalence in employees interacting with the public versus all other employees in Study 1.

				% K6 in moderate to high range		
					
Sector	Total N Employees	Employee category	% customer staff	Customer staff	Non customer staff	P
Public	1472	Customer services	23.3%	14.7%	14.7%	0.518
Private	1113	Contact centre	7.4%	21.7%	14.4%	0.06
Private	281	Call centre	9.4%	14.8%	10.2%	0.46
Private	1627	Customer service	19.8%	24.8%	17.2%	0.002
Private	1043	Customer services	14.9%	12.8%	11.3%	0.58
Public	5723	Counter staff	80.1%	20.0%	17.3%	0.035

In 2007 a second survey (including the HPQ and the K6) was distributed via the internet to the sales staff of a leading Australian bank (Study 2). This survey also questioned respondents on the number of interactions they had with the public in the previous week. In total 7,179 employees were invited to respond to the initial questionnaire of which 30% (N = 2,129) responded (67% female; mean age 39-years SD 10-years). Demographic variables recorded by the HPQ included as covariates for subsequent ANOVA comparisons are gender, age (18-24, 25-34, 35-44, 45-54, 66-64, 65 and over), marital status (married or cohabiting, separated, divorced, widowed and never married) and education level (less than year 10, year 10, year 12, some tertiary education, degree graduate, postgraduate degree).

The research was carried out in compliance with the Helsinki Declaration. The University of Queensland Human Research Ethics Committee approved the study protocol (Clearance number 2004000304).

### The Kessler 6 (K6)

The K6 is a six-item scale of psychological distress that strongly discriminates between those who meet and do not meet diagnostic criteria for a mental disorder[24-27]. The K6 has excellent internal consistency with Chronbach's alpha of 0.89[24]. In this study we employed published methods where a K6 score of 0 to 7 reflects low psychological distress, a score of 8-12 indicates moderate psychological distress and a score of 13 to 24 represents high psychological distress[25,26].

### Statistics

In data from the WORC project (Study 1) comparison of the prevalence of moderate or high psychological distress by employees who do and do not interact with the public was performed using Pearson's χ^2 ^statistic for each separate employer and the data set as a whole. These results are considered indicative as they do not account for covariates. To determine the odds ratio (OR) that interaction with the public may result in moderate to high psychological distress a binomial multivariate model with the outcome measure being psychological distress (low psychological distress versus moderate or high psychological distress) was run with customer contact (yes or no) as a covariate and simultaneous additional covariates of sex, age, level of education and marital status. To further explore the relationship between psychological distress and interaction with the public a multinomial logistic regression was performed with K6 category as the dependent variable and K6 score of low psychological distress as the reference category. For the multinomial logistic regression the factor of customer contact "Yes" or "No" was examined while using covariates of gender, age, marital status and education level. In the data from sales staff in the Australian bank (Study 2), number of customer contacts was recorded. A univariate analysis of variance (ANOVA) was performed with psychological distress score (range 0 to 24) as the dependent variable, number of customer contacts per week (categorised into 0 -24, 25 - 49, 50 - 89, 91 -149 and 150+) was the fixed factor with covariates of sex, age, level of education and marital status.

All statistics were performed using SPSS Version 17.0.

## Results

In the WORC sample (study 1) the mean age of both the contact with the public and those without contact with the public was 40-years old with no significant difference between the groups. The gender distribution was significantly (χ^2 ^< 0.001) different with those in contact with the public having 66% females whilst those without contact with the public had 48% females. Table 1 describes the prevalence of moderate or high psychological distress in employees defined as interacting with the public versus all other employees (Study 1). What can be noted from Table 1 is that in two of the six employers the prevalence of psychological distress is significantly higher in employees that interact with the public. In one employer the difference approached statistical significance (P = 0.06). If the data from all six employers is combined the prevalence of psychological distress in customer staff is 19% where as in non-customer staff the psychological distress prevalence is 15%. This difference is statistically significant (P < 0.001). These results are indicative that interacting with the public may be associated with increased levels of psychological distress.

The above χ^2 ^analysis does not take into account the influence of demographic variables on the presence or absence of psychological distress. Table 2 presents the results of binomial logistic regression with demographic variables simultaneously included in the model along with interaction with the public. Interacting with the public significantly (P < 0.001) elevates the OR for moderate to high psychological distress to 1.3 once accounting for demographic covariates. Table 3 presents the results of the multinomial logistic regression. It can be noted from table 3 that customer contact significantly increases the OR for moderate psychological distress to 1.13 and for high psychological distress to 1.33. This indicates that customer contact is a greater risk factor for high psychological distress than it is for moderate distress. In both the binomial and the multinomial logistic regression gender was not a significant factor influencing the OR for psychological distress (p = 0.285 for binomial regression and p = 0.369 and 0.146 for moderate and high psychological distress in multinomial regression).

**Table 2:** Binomial logistic regression results with moderate or high psychological distress as the dependent variable for Study 1

		**95% C.I**.		
	Odds ratio	Lower	Upper	**Sig**.
Customer contact = yes	1.29	1.13	1.47	.000
Age	0.98	0.98	0.99	.000
Gender (female)	1.07	0.94	1.21	.285
				
**Marital Status**				
Married or cohabitating				.000
Separated	2.13	1.67	2.73	.000
Divorced	1.48	1.19	1.84	.000
Widowed	1.54	0.86	2.77	.145
Never married	1.22	1.05	1.42	.009
				
**Education attainment**				
< Year 10				.000
Year 10	0.77	0.52	1.15	.198
Year 12	0.60	0.40	0.90	.013
Some tertiary	0.60	0.41	0.88	.008
Degree graduate	0.49	0.33	0.73	.000
Post graduate degree	0.41	0.27	0.64	.000
Constant	0.53			.000

**Table 3:** Multinomial logistic regression results with K6 category as the dependent variable and K6 score of low psychological distress as the reference category

			95% CI		
K6 Category		OR	Lower Bound	Upper Bound	**Sig**.
Moderate Distress	**Gender**				
	Male	0.97	0.90	1.04	0.369
	Female	Reference Category			
	**Age**	0.98	0.98	0.98	.000
	**Marital Status**				
	Married or cohabitating	0.79	0.73	0.86	< 0.001
	Separated	1.43	1.19	1.71	< 0.001
	Divorced	1.03	0.89	1.20	0.682
	Widowed	0.36	0.82	1.72	0.355
	Never Married	Reference category			
	**Education**				
	< year 10	2.12	1.63	2.76	< 0.001
	Year 10	1.29	1.11	1.50	0.001
	Year 12	1.28	1.11	1.47	0.001
	Some tertiary	1.26	1.11	1.42	< 0.001
	Degree graduate	1.21	1.06	1.38	0.004
	Post graduate degree	Reference category			
	**Customer contact**				
	Customer contact = Yes	1.13	1.05	1.21	0.002
	Customer contact = No	Reference category			.

High Distress	**Gender**				
	Male	0.91	0.81	1.03	0.146
	Female	Reference category			
	**Age**	0.98	0.98	0.98	.000
	**Marital Status**				
	Married or cohabitating	0.65	0.56	0.75	< 0.001
	Separated	2.08	1.63	2.67	< 0.001
	Divorced	0.95	0.74	1.22	0.701
	Widowed	0.82	0.41	1.61	0.560
	Never Married	Reference category			
	**Education**				
	< year 10	2.54	1.63	3.98	< 0.001
	Year 10	1.98	1.52	2.58	< 0.001
	Year 12	1.50	1.16	1.95	0.002
	Some tertiary	1.59	1.26	2.01	< 0.001
	Degree graduate	1.14	0.89	1.47	0.298
	Post graduate degree	Reference category			
	**Customer contact**				
	Customer contact = Yes	1.33	1.17	1.51	< 0.001
	Customer contact = No	Reference category			.

In the sample from the sales employees of the large Australian bank (Study 2) 18.7% of employees had either moderate or high psychological distress. Table 4 contains the results of the ANOVA with K6 score as the dependent variable. This data indicates that all categories where customer contacts are > 24 per week are significantly different (P < 0.016 for all comparisons) from employees with less than 24 or less customer contacts per week. There are no statistical differences between customer contact groups above the 24 contacts per week. This data indicates that customer contact above 24 contacts per week universally elevates psychological distress.

**Table 4:** Study 2 ANOVA results for K6 score (dependent variable) and customer contacts (fixed factor).

			95% CI	
			
Contact Number	Mean K6 score	Std. Error	Lower	Upper
0 to 24	3.7	0.4	2.9	4.6
25 to 49*	5.3	0.4	4.5	6.1
50 to 89*	5.1	0.3	4.4	5.8
90 to 149*	5.3	0.4	4.6	6.1
150+*	5.2	0.4	4.5	5.9

Caption: * indicates group is significantly different from the reference group of 0 - 24 contacts. There are no statistical differences between the groups of 25 - 49, 50 - 89, 90 - 149 and 150+ contacts.

## Discussion

It is thought that employees such as nurses[12-15], teachers[16-19], call centre operators[20,21] and sales staff [3,22], are at greater risk for psychological distress. We hypothesised that occupations that require employees to interact with the public may pose an increased risk of psychological distress.

From the original WORC data (Study 1) the comparisons of psychological distress by whether employees interact with the public or not (Table 1) raises the possibility that interacting with the public increases the prevalence of psychological distress over employees that do not interact with the public. A limitation of this finding may be that employee categories other than customer services, contact centre staff or counter may have also interacted with the public. If this conjecture is true, it would likely lead to a reduction in the difference between the defined categories of interacting with the public and all other staff. Binomial logistic regression results in table 2 confirm that interacting with the public significantly increases the OR for moderate or high psychological distress. Multinomial logistic regression results in table 3 indicate that customer contact significantly increases the OR for moderate and high psychological distress. Of interest here is that contact with the public has a greater effect on the OR for high psychological distress.

Study 2 examines psychological distress by number of customer contacts data from sales staff of a bank. Table 4 suggests that any interaction with the public of 25 contacts per week and above increases the mean psychological distress score. It may be that interaction with the public per se does not predict psychological distress it may in fact be that the number of difficult contacts is the predictor. The total number of contacts may be a surrogate for difficult contacts as the more contacts an employee has the greater the risk of a difficult contact. How employees, by nature or by training, are able to deal with encountering a member of the public that is difficult or abusive is not measured in this study. Employees who have more difficulty dealing with the emotions potentially arising from having to deal with difficult members of the public would be expected to show more psychological distress.

Due to the relative large numbers of employees surveyed the results that interaction with the public increases psychological distress may be extrapolated to other industries not surveyed in this study. However, interaction with the public has different forms and may not be equal across occupations. For example a nurse interacting with the public likely has different components to the interaction than a sales person. It may be more likely that a nurse is respected in client interactions than a sales person or call centre operator. How various occupations change the relationship between contact with the public and psychological distress is an area where further research could focus.

## Limitations

A limitation of this data is that all employees surveyed in the bank study (Study 2) were identified as sales staff and all except 13 of these had interactions with the public. Thus there was no group of substantial size of employees who did not interact with the public for comparison. Additionally, as mentioned above, sales staff have an increased risk of psychological distress. In the bank sample all respondents were sales staff and there may be aspects of sales, other than interacting with the public, that increase the risk of psychological distress. These potential other factors may serve to dilute the ability to detect public interaction as a primary predictor of psychological distress in the sales force.

A strength of the study is the large sample size however the sample of employees was not randomly selected. Companies (employers) self-selected as to whether they would participate in the project. Employees from the companies self-selected as to whether they would respond to the survey. However, self-selection biases in the current data are representative of those inherent in any employee health screening survey. Companies self-select as to whether the run a HRA survey and employees are free to choose whether they respond. Consequentially the current paper represents phylogenetically valid methodology. The response rate of 25% to the HPQ survey is low in epidemiological terms. However, this is the typical response rate obtained when health questionnaires are sent to employees in multiple large employers[28-31]. Although the response rate is typical it may not be representative. Comparison of responders to non-responders, to a questionnaire containing mental health questions, showed no statistical difference in the Hamilton Depression Scale[32]. Previous studies indicate that a variation in the response rate is not related to prevalence of chronic conditions (including depression)[29]. Additionally in the data set we present here we have previously shown that response rates are not predictive of prevalence of psychological distress[22].

The logistic and multivariate regressions in Study 1 and the ANOVA in study 2 included socio-demographic variables as covariates. In addition to socio-demographic variables health behaviours of employees could partly explain the relationship between interacting with the public and psychological distress. The health questionnaire applied, the HPQ, does not record data on health behaviours. This is an area where further research could focus.

Previous research has identified that an effort-reward imbalance or decreased job control and increased job demands may lead to increasing psychological problems in employees. It may be that occupations that interact with the public have alterations in these job characteristics. These variables were not part of the survey applied, the HPQ. Further work on employee psychological distress and interaction with the public may benefit by inclusion of job control-demands and/or effort-reward explanatory variables.

For study 1 intensity/amount of interaction with the public is not known in this sample. As in study 2, presented in this paper, future studies could include the number of interactions with the public or the amount of time spent interacting with the public as predictors of psychological distress. As the data presented is cross sectional, causality between psychological distress and interaction with the public cannot be definitively established. For example it is possible that a psychological distress my produce increased problems when interacting with the public.

## Conclusions

The results of the current study support the contention that interaction with the public increases levels of psychological distress in employees. Further research is required to elicit the characteristics of employees that make them vulnerable to respond with psychological distress to certain interactions with the public and to better understand how to provide employee training and support programs that will assist to prevent or mitigate this distress.

## Abbreviations

ANOVA: Analysis of variance; HPQ: Health and Work Performance Questionnaire; HRA: Health risk appraisal survey; K6: Kessler 6 scale of psychological distress; NCS: US National Comorbidity Survey; WORC: Work Outcomes Research Cost-benefit Project.

## Competing interests

The authors declare that they have no competing interests.

## Authors' contributions

MH contributed to the conception and design of the research, participated in the acquisition of data, performed the statistical analyses, interpreted the data, drafted the manuscript and approved the final version to be published. HW contributed to the conception and design of the research, interpreted the data, assisted in drafting the manuscript and approved the final version to be published.

## Pre-publication history

The pre-publication history for this paper can be accessed here:

http://www.biomedcentral.com/1471-2458/10/435/prepub
